# Sol–Gel Synthesized Silica/Sodium Alginate Hybrids: Comprehensive Physico-Chemical and Biological Characterization

**DOI:** 10.3390/molecules30173481

**Published:** 2025-08-25

**Authors:** Antonio D’Angelo, Cecilia Mortalò, Lara Comune, Giuseppina Raffaini, Marika Fiorentino, Michelina Catauro

**Affiliations:** 1 Department of Engineering, University of Campania “Luigi Vanvitelli”, Via Roma 29, 81031 Aversa, Italy; antonio.dangelo@unicampania.it; 2 Institute of Condensed Matter Chemistry and Technologies for Energy (ICMATE-CNR) National Research Council (CNR), C.so Stati Uniti 4, 35127 Padova, Italy; cecilia.mortalo@cnr.it; 3 Department of Environmental, Biological and Pharmaceutical Sciences and Technologies, University of Campania “Luigi Vanvitelli”, Via Vivaldi 43, 81100 Caserta, Italy; lara.comune@unicampania.it; 4 Department of Chemistry, Materials, and Chemical Engineering ‘‘Giulio Natta’’, Politecnico di Milano, Piazza L. da Vinci 32, 20131 Milan, Italy; giuseppina.raffaini@polimi.it

**Keywords:** SiO_2_, sodium alginate, sol–gel, surface area, BET, thermal analysis, moisture, cytotoxicity

## Abstract

The development of biomaterials with tailored properties is indispensable for biomedical applications. In this study, amorphous silica/sodium alginate (SiO_2_/SA) hybrids were synthesized via the sol–gel method by incorporating 2, 5, and 8% sodium alginate into the silica matrix. The hybrids were characterized to evaluate their structural, surface, thermal, moisture-responsive, and biological properties. FTIR and XRD analyses confirmed the formation of organic–inorganic networks and amorphous structures. BET measurements revealed a specific surface area of 325 m^2^/g for SiO_2_/SA2%, decreasing with higher SA content to 104.3 m^2^/g for SiO_2_/SA8%; the moisture sorption capacity followed a similar trend. Thermal analysis indicated improved stabilization of the polymer within the silica matrix. Cytotoxicity tests on HaCaT (human keratinocyte) cells line revealed moderate toxicity for the SiO_2_/SA2% hybrid (~40% cell viability inhibition (CVI)), while increasing the SA content reduced cytotoxicity, with a CVI of 33% for SiO_2_/SA5% and ~15% for SiO_2_/SA8%, all within non-toxic ranges according to ISO standards. The SiO_2_/SA5% hybrid demonstrated the best balance between functional properties and biocompatibility. These preliminary results suggest that further optimization with intermediate SA concentrations (e.g., 6–7%) could further reduce cytotoxicity while maintaining desirable properties, supporting the potential of silica/sodium alginate hybrids in future biomedical applications.

## 1. Introduction

Biomaterials are a class of materials which are able to interact with a specific part of the human body to enhance and assist tissue regeneration. Indeed, their interactions with damaged tissues stimulate growth and repair [1,2,3]. Nowadays, several biomaterials, including natural and synthetic polymers, metal oxides, and bioceramics, provide flexible solutions for tissue regeneration and drug delivery. According to their composition, they are divided into four categories: (i) metallic, (ii) ceramic, (iii) polymers, and (iv) biocomposites [4,5]. Among ceramics, amorphous silica represents a valuable choice as a biomaterial due to its chemical inertness, which prevents undesirable reactions with surrounding molecules. Moreover, it exhibits favorable surface properties, such as a well-developed surface area and numerous hydroxyl groups on its surface. Unlike bioceramics obtained through traditional melting process, sol–gel-derived glasses are produced at lower temperatures, resulting in higher surface area and porosity [6,7,8]. This improves bioactivity [9], bone bonding [10,11], drug release [12], and degradation rates [13].

The abundance of –OH groups on the silica surface enables hydrogen bonding with various pharmacologically relevant molecules, which can be adsorbed onto its porous structure, making it highly suitable for drug delivery applications [14]. In addition, the high surface area of amorphous silica contributes to its biomimetic potential and biocompatibility [15]. The rough surface topography also facilitates cell attachment and the proliferation of stem cells, which play a key role in effective tissue regeneration, promoting better integration with biological tissues [16,17].

The incorporation of a natural organic polymer into the silica matrix improves biocompatibility and increases the tolerance within the host tissue. It also enhances the degradability of the material, potentially eliminating the need for a further surgical procedure to remove the material once the tissues have healed and regenerated [18,19].

Numerous studies have highlighted that incorporating natural polymers into the inorganic silica matrix can significantly improve the properties of silica-based biomaterials. For instance, the combination of chitosan with silica to form xerogels has been shown to exhibit a high surface area, bioactivity, and osteoconductive properties, thereby enhancing cell differentiation and prompting quick bioactive responses [20]. Similarly, a silica/collagen system synthesized via the sol–gel method provided a fiber-reinforced, porous structure with mechanical properties making it applicable for bone proliferation in low-load-bearing areas of body [21]. Other hybrids have been synthesized by combining silk fibroin with silica, improving their biomedical applicability [22]. Another organic polymer of biomedical interest is alginate, a polysaccharide obtained from cell walls of brown algae and bacterial capsules of *Azotobacter* sp. and *Pseudomonas* sp. The most common form is sodium alginate [23].

Alginates are composed of alternating β-D-mannuronic acid (M) and α-L-guluronic acid (G) units which are linked by 1,4-glycosidic bonds. Alginates with a high M content form more elastic, non-toxic, and non-immunogenic hydrogels, making them more biocompatible, more degradable, and highly suitable for soft tissue regeneration and controlled drug release [18,24]. The incorporation of such biopolymers increases the biocompatibility of silica-based biomaterials and improves their structural properties, particularly the swelling capacity, allowing them to absorb body fluids without undergoing structural collapse, thereby increasing their adaptability within human tissues [18,25,26,27].

Since most of the recent literature deals with hybrid materials in which silica nanoparticles are embedded in alginate beads or composites [28,29], the aim of this work was to synthesize SiO_2_/sodium alginate (SA) hybrid materials by incorporating 2, 5, and 8% of the polymer via the sol–gel route to obtain biomaterials with enhanced properties for potential applications in the biomedical field. The hybrids were characterized in terms of their chemical composition using FTIR, their structural features through XRD analysis, their surface area via BET analysis, as well as their thermal behavior and moisture sorption capacity. Biocompatibility was assessed by testing the hybrids against HaCaT cell cultures, which are used as a model of healthy human cells. The goal was to obtain preliminary data to evaluate how the incorporation of sodium alginate can improve the properties of amorphous SiO_2_.

## 2. Results and Discussion

### 2.1. Sol–Gel Synthesis

**Figure 1:**
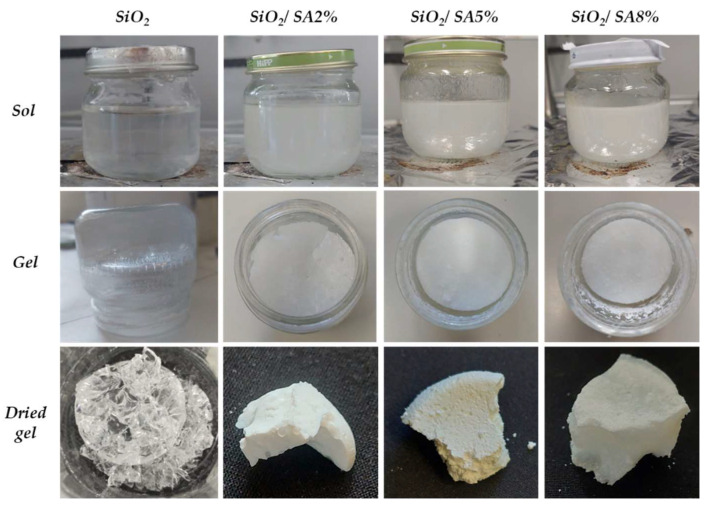
SiO_2_/SA hybrids images compared to SiO_2_ in sol, gel, dried gel steps.

The SiO_2_/SA hybrids, as well as the pure SiO_2_, were synthesized using the sol–gel technique through hydrolysis and polycondensation reactions in the presence of water and methanol. Methanol was selected as a co-solvent due to its relatively high polarity. This polarity prevents the precipitation of sodium alginate, which is unstable in the presence of long-chain alcohols such as ethanol. The synthesis was conducted under acidic conditions to accelerate the hydrolysis reactions relative to the condensation steps. An acidic environment promotes the hydrolysis of the tetraethyl orthosilicate (TEOS) precursor by facilitating the cleavage of alkoxy (–OR) groups from the water molecules and leads to the formation of numerous silanol (–Si–OH) groups. The subsequent delay in the condensation of these silanol groups, caused by the protonation of the hydroxyl moieties, results in the formation of a limited number of nuclei. These nuclei then grow into large amorphous silica bulks, characterized by a dense matrix with a fine and narrowly distributed porosity [30]. The incorporation of sodium alginate (SA) significantly modified this process. Under acidic conditions, the carboxylate groups (–COO^−^) of SA were protonated to –COOH, losing their negative charge and becoming capable of forming hydrogen bonds with the silanol groups (–Si–OH) generated from TEOS hydrolysis, the SiO_2_ precursor of these sol–gel synthesis [23]. These hydrogen bonds promoted the formation of an organic–inorganic hybrid network and locally inhibited or modulated the condensation of the silica species. Additionally, SA adopts a highly hydrated and expanded conformation in aqueous solution due to its polyelectrolyte nature. During the early stages of silica condensation, this polymer network swells, taking up space and sterically hindering the formation of compact silica agglomerates [31]. As a result, preliminary morphological observations based on the acquired digital photographs of dried SiO_2_/SA hybrids showed spongy-like structures, potentially indicating a more open porosity with respect to pure SiO_2._ In addition, hybrids lost the typical transparency of SiO_2_ glass, appearing instead as opaque and white materials (Figure 1).

### 2.2. FTIR Analysis

**Figure 2:**
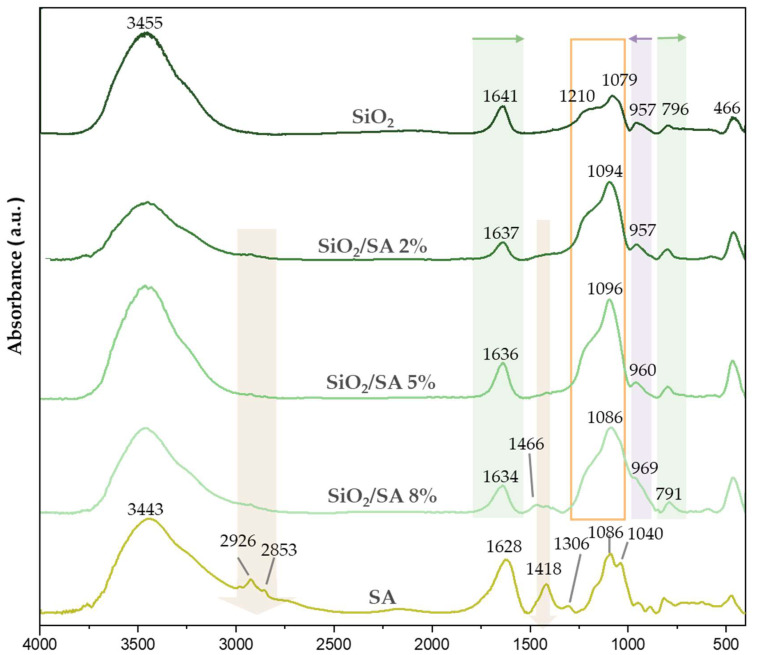
FTIR spectra of SiO_2_/SA hybrids. Pink arrows indicate increasing peak intensity; green filled squares indicate red shifts; violet filled squares indicate blue shifts; orange empty squares highlight the complex Si–O–Si band region.

An FTIR study was performed to investigate the interaction between the SiO_2_ network and sodium alginate (SA) polymer during the formation of the hybrid materials. The FTIR spectra of SiO_2_/SA hybrids, pure SiO_2_, and pure SA are shown in Figure 2. In the spectrum of pure sodium alginate, the broad band centered at 3443 cm^−1^ was attributed to O–H stretching vibrations of hydroxyl groups, while the sharp peaks at 2926 cm^−1^ and 2852 cm^−1^ corresponded to the C–H stretching vibrations of methylene (–CH_2_–) groups in the saccharide backbone [32,33]. The peaks at 1628 cm^−1^ and 1418 cm^−1^ were assigned to the asymmetric and the symmetric stretching vibrations of carboxylate (νCOO^−^) groups, respectively [34,35]. The band at 1308 cm^−1^ was attributed to C–O stretching. Meanwhile, the peaks at 1086 cm^−1^ and 1040 cm^−1^ were associated with νC–O and νC–O–C stretching modes, linked to mannuronic (M) and guluronic (G) units [36,37]. These features, particularly the C–H stretching and symmetric νCOO^−^ peaks, were still visible in the spectra of hybrid samples and increased in intensity with SA content (as indicated by pink arrows in Figure 2). In pure SiO_2_, the broad band centered at 3455 cm^−1^ and the peak at 1641 cm^−1^ were attributed to O–H stretching and bending vibrations, respectively, from silanol groups. The 1641 cm^−1^ band underwent a red shift to 1637 cm^−1^ in the SiO_2_/SA 8% hybrid, suggesting the formation of hydrogen bonds between silanol groups and polysaccharide hydroxyls [38,39]. The broad band at 1079 cm^−1^ and the peak at 796 cm^−1^ in pure silica corresponded to Si–O–Si asymmetric and symmetric stretching vibrations, respectively [40]. The 796 cm^−1^ band showed a gradual red shift with increasing SA, reaching 791 cm^−1^ in the 8% hybrid. Conversely, the Si–O–Si asymmetric band shifted toward higher wavenumbers (i.e., a blue shift), reaching ~1096 cm^−1^ in the 8% SA hybrid. This shift indicated increased network rigidity; however, this likely arose from the physical entrapment of excess SA rather than additional hydrogen bonding. The non-interacting polymer may have induced the formation of SA-rich heterogeneous domains, leading to a structurally less uniform and thermodynamically less stable hybrid material. Additionally, its shape changed significantly, suggesting overlapping contributions from both Si–O–Si vibrations and carboxylate bands at ~1086 and 1040 cm^−1^. The increasing intensity and complexity of this band may also have been due to the growing presence of –COO^−^ groups from SA overlapping the Si–O–Si signal.

**Figure 3:**
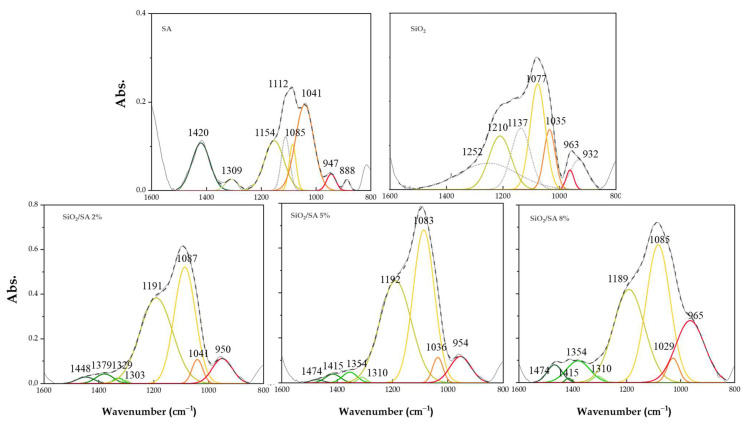
Deconvoluted spectra of SiO_2_/SA hybrids in the range of 1600–800 cm^−1^ compared to pure SiO_2_.

**Figure 4:**
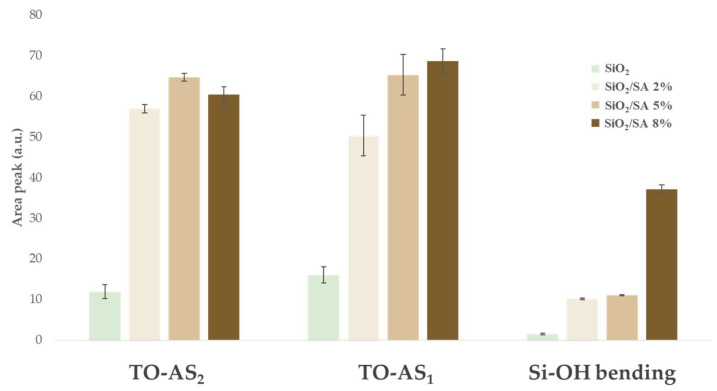
Area peak of deconvoluted Si-O-Si band in the range 1600–800 cm^−1^ of SiO_2_/SA hybrids in the range of 1600–800 cm^−1^ compared to SiO_2._ TO-AS_2_: 1210 cm^−1^ peak; TO-AS_1_: 1077–1085 cm^−1^ peak.

To further investigate the impact of the polymer on the silica network, peak deconvolution was performed in the 1600–800 cm^−1^ region of the Si-O-Si stretching band. Pure SiO_2_ displayed seven deconvoluted peaks at approximately 1252, 1210, 1137, 1077, 1035, 963, and 932 cm^−1^, which were attributed to the asymmetric stretching modes of the transverse optical (TO) siloxane framework [41]. According to De los Arcos et al. [41], the peaks at 1137 and 1210 cm^−1^ (both associated with TO–AS_2_ modes) reflect different structural features: the 1137 cm^−1^ band is directly related to terminal silanol groups (Si–OH) which are present on the surface or within porous domains, indicating reactive sites and surface defects. The 1210 cm^−1^ peak corresponds to asymmetric Si–O–Si vibrations and is typically used as a marker of topological disorder and reduced network connectivity. The bands in the 1035–1077 cm^−1^ region (TO–AS_1_) are attributed to more ordered and crosslinked Si–O–Si domains, while the lower-frequency peaks at 963 and 932 cm^−1^ are associated with silanol bending and stretching modes, further indicating structural defects and surface hydroxylation [41]. SA exhibited characteristic saccharide bands that partially overlapped with the Si–O–Si stretching region: 1154 and 1112 cm^−1^ (asymmetric and symmetric C–O–C stretching), 1085 and 1041 cm^−1^ (C–O and C–O–C vibrations from mannuronic/guluronic units), and 947 and 888 cm^−1^ in the anomeric region, associated with glycosidic bond vibrations and deformation modes [42]. The deconvolution analysis of the SiO_2_/SA hybrids revealed significant spectral modifications, indicating molecular interactions and structural rearrangements (Figure 3). The 1137 cm^−1^ band (grey curve in Figure 3), which is associated with terminal Si–OH groups in pure SiO_2_, disappeared in the hybrid samples, suggesting a chemical interaction with the –COOH groups of sodium alginate. Additionally, the 1210 cm^−1^ band (green curve in Figure 3) was red shifted to approximately 1191 cm^−1^ upon hybrid formation, accompanied by increased intensity. This shift indicated enhanced structural disorder induced by SA incorporation. The area of the TO–AS_2_ band (initially centered at 1210 cm^−1^) increased with increasing SA content, reaching a maximum in the SiO_2_/SA 5% sample and decreasing slightly at 8%. This was likely due to a redistribution of vibrational modes and the growing influence of the organic phase. The 1085 cm^−1^ band (yellow curve in Figure 3) also showed increased intensity with higher SA content due to overlapping contributions from the 1077 cm^−1^ SiO_2_ band and the 1085 cm^−1^ saccharide band of SA. This TO–AS_1_ contribution increased from 15 to 68 of the total area (Figure 4) due mainly to the increased SA amount. Finally, the 963 cm^−1^ band (red curve in Figure 3) increased significantly in intensity in the hybrid materials and correlated with the SA concentration. Its integrated area increased fourfold compared to pure SiO_2_, further confirming the incorporation of organic content (Figure 3). Although primarily associated with Si–OH bending vibrations, this band likely included contributions from glycosidic bonds and suggested that the alginate structure had remained intact despite the acidic conditions. This indicated a protective effect of the silica matrix, which likely shielded the polymer from acid hydrolysis during hybrid formation [23].

### 2.3. XRD Analysis

**Figure 5:**
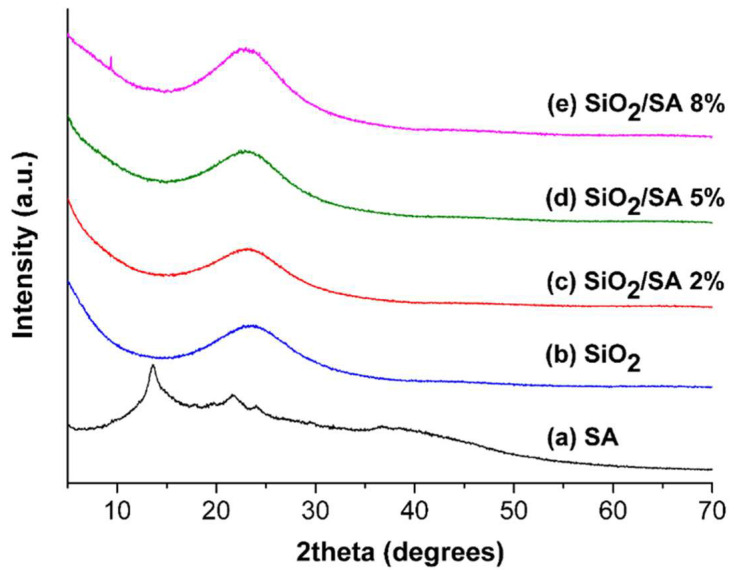
XRD profiles of pure SA and SiO_2_ and SiO_2_/SA hybrids materials prepared by sol–gel method.

Figure 5 shows the XRD patterns of pure sodium alginate (SA) and silica (SiO_2_), as well as the SA/SiO_2_ hybrid materials prepared by the sol–gel method. The diffractogram of the commercial sodium alginate powders (Figure 5a) exhibited two broad signals centered at 13.6° and 21.8°. These peaks were associated with the semi-crystalline arrangement of guluronate and mannuronate units in the commercial SA sample [43,44,45]. An additional peak at 24° was also attributable to the sodium alginate structure, as observed in recent studies [46,47]. However, these signals were no longer visible in the SA/SiO_2_ hybrid samples, including those with an SA composition of 8%. Indeed, the XRD patterns of the SA/SiO_2_ hybrids (Figure 5c–e) showed amorphous structures similar to pure SiO_2_ (Figure 5b). A broad peak around 23° indicated low crystallinity. The absence of the SA peaks in the XRD patterns of the hybrid materials was likely due to the polymer’s amorphization during the sol–gel process, as well as its low content and dispersion within the silica matrix. In the hybrid materials, SA was embedded within a continuous silica matrix and constituted only 8 wt% of the total mass. This low content, combined with molecular-level dispersion within the inorganic network, reduced the intensity of any residual SA diffraction signals further, especially those not overlapping with the silica halo. These factors hindered the formation of detectable crystalline domains. Although the main diffraction patterns of the SA/SiO_2_ hybrids resembled those of pure amorphous silica, subtle differences could be observed. The hybrids exhibited a slightly noisier background and a shallower negative slope at low 2θ values (below 17°) than pure SiO_2_. Samples with a higher SA content revealed a faint, broad feature at around 13.6°, which may have resulted from residual short-range ordering of the guluronate and mannuronate portions of the original polymer structure. Additionally, the intensity of the broad peak around 24° increased with higher SA content, potentially due to a partial overlap between the silica and the most intense sodium alginate reflection. These minor variations were consistent with the progressive increase of the organic phase within the silica matrix. However, they did not indicate the presence of detectable crystalline domains. Furthermore, the narrow peaks observed around 10° only in the 8% SA sample may have originated from minor crystalline by-products or residual salts introduced by the sodium alginate source or formed during processing. Further analysis will be conducted to determine their exact origin. This result is consistent with other SiO_2_-based hybrid materials prepared by the sol–gel method, where crystalline organic polymers or drugs were incorporated, as reported in other studies [48,49].

### 2.4. BET Study

Table 1 reports the specific surface area values determined by the Brunauer-Emmett-Teller (BET) method for samples pre-treated at 80 °C for 24 h. BET analysis involves the adsorption of nitrogen gas onto the sample surface at low temperatures. The gas molecules form a monomolecular layer, and by knowing the diameter of the nitrogen molecule and the number of molecules required for complete coverage, the total surface area of the sample can be calculated. As shown in Table 1, the specific surface area of the as received SA was very low (0.2307 m^2^/g), which was consistent with its semicrystalline nature observed in the XRD analyses. In contrast, the bare SiO_2_ gel exhibited a significantly higher surface area value (9.6861 m^2^/g), typical of its amorphous structure. Interestingly, a synergistic effect between SA and SiO_2_ was observed: the specific surface area of the SiO_2_/SA hybrid gels increased by more than one order of magnitude compared to pure SiO_2_. The highest BET surface area (325.2 m^2^/g) was recorded for the SiO_2_/SA 2% sample, while the surface area decreased to 138.7 and 104.3 m^2^/g in hybrids containing 5 and 8% SA, respectively. The initial increase in surface area with the addition of a small amount of SA (2%) could be attributed to the highly hydrated and expanded conformation of alginate in aqueous solution, which formed a swollen polymer network, as previously discussed in Section 2.1. The weak interaction between the alginate and the silica matrix prevented shrinkage or structural collapse during gelation and drying, promoting the formation of a more open structure with a greater accessible surface area. However, at higher SA content (5 and 8%), the surface area decreased by approximately two-fold and three-fold, respectively. This can be explained by the increasing of SA filling the voids within the silica network, reducing the accessible surface, and altering the hybrid gel structure. These results are inconsistent with those reported by Han et al. for sodium alginate-silica composite aerogels prepared via sol–gel process [50]. In their study, it was observed that increasing the sodium alginate content led to a gradual thickening of the composite aerogel cell walls, hindering pore formation and decreasing pore volume.

### 2.5. Moisture Sorption Analysis

**Figure 6:**
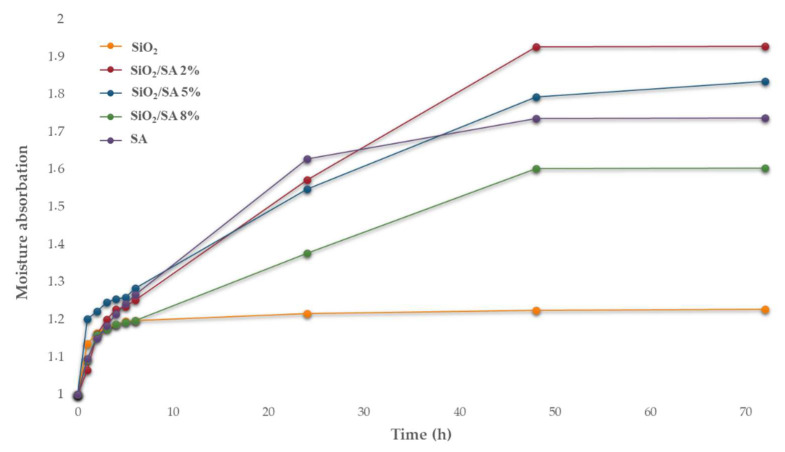
Moisture absorption test of SiO_2_/SA hybrids underwent 99% relative humidity.

A moisture absorption test was conducted on the hybrid materials to evaluate the effect of incorporating SA on the moisture sorption capacity of SiO_2_, which is well known for its hydrophilicity due to the large number of hydroxyl groups on its surface [51]. This property is particularly relevant for biomaterials designed for cell and tissue contact, as the ability to retain moisture improves biocompatibility, supports cell viability, and facilitates integration within hydrated biological environments [52]. The moisture sorption graphs show the ratio of the weight at various time points to the initial weight of different samples exposed to 99% relative humidity, reflecting their moisture uptake behavior (Figure 6). Pure SiO_2_ exhibited relatively limited moisture absorption, reaching a weight ratio of approximately 1.23 after 72 h. Conversely, pure sodium alginate (SA) exhibited significantly higher moisture uptake, reaching a weight ratio close to 1.73. SiO_2_/SA hybrid materials displayed intermediate moisture absorption characteristics: higher than pure SiO_2_ but generally lower than pure SA. Notably, the hybrids containing 2% and 5% SA demonstrated the highest moisture uptake, approaching a weight ratio of 1.9 at 72 h. In contrast, the 8% SA hybrid absorbed less moisture relative to these two but more than pure SiO_2_. These observations correlated strongly with the BET-specific surface area measurements. The hybrids with 2% and 5% SA possessed substantially increased surface areas (approximately 325 m^2^/g and 139 m^2^/g, respectively) compared to pure SiO_2_ (≈9.7 m^2^/g) and pure SA (≈0.23 m^2^/g). This indicated enhanced mesoporosity and a greater available surface for interaction with water molecules, facilitating higher moisture uptake. The reduced moisture absorption of the 8% SA hybrid aligns with its lower surface area compared to the 2% and 5% hybrids, suggesting that the increased SA content began to fill the open pores of the hybrid matrix, thereby reducing the availability of sites for moisture uptake.

### 2.6. Thermal Analysis

**Figure 7:**
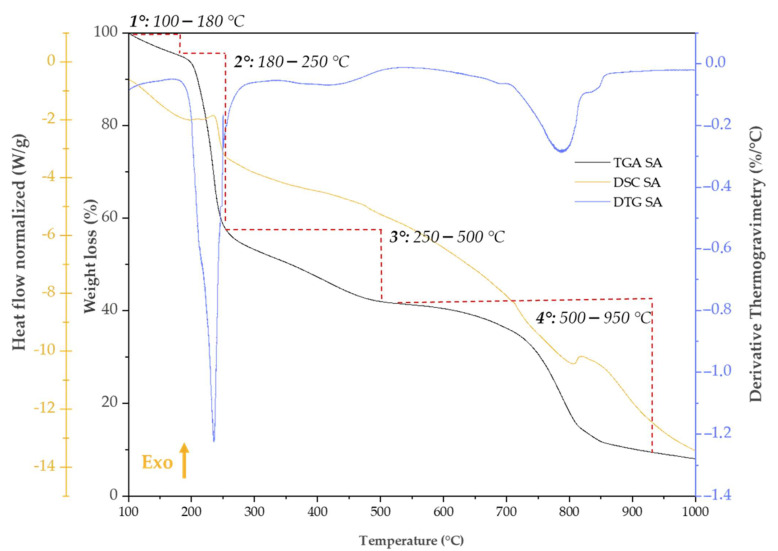
TGA, DSC, and DTG curves of pure sodium alginate. Weight loss of TGA curves is underlined with red dashed curve.

Thermal analysis was carried out to assess the thermal stability of sodium alginate (SA) within the SiO_2_/SA hybrid materials under nitrogen atmosphere. The TGA/DSC curve of pure SA (Figure 7) showed four main weight loss events. The first stage, from 100–180 °C, was due to the evaporation of absorbed water, with a weight loss of 4.83%; the second stage, from 200 °C to 250 °C, showed a further weight loss of about 40.34%, associated with the thermal decarboxylation of uronic acid residues [53] and cleavage of the glycosidic backbone [54,55,56]. This was accompanied by an endothermic peak in the DSC curve. In the second stage (250–500 °C), a further 13.40% weight loss occurred, attributed to carbonization of the polymer matrix and the formation of sodium carbonate (Na_2_CO_3_). Finally, between 500 °C and 950 °C, a further 30.81% mass loss was observed due to the decomposition of Na_2_CO_3_ into sodium oxide (Na_2_O) and CO_2_, as confirmed by the DTG peak at 786.78 °C [57,58,59].

**Figure 8:**
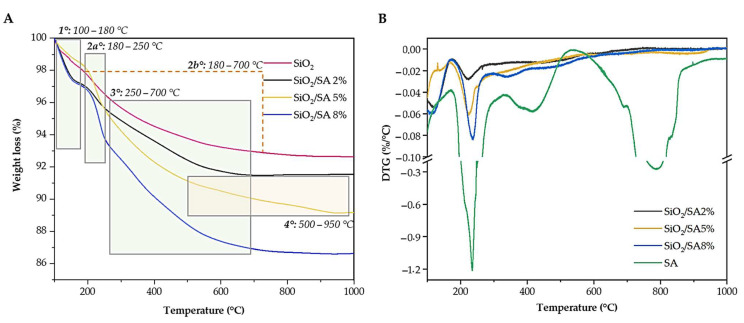
(**A**) TGA curves of SiO_2_/SA hybrids; (**B**) DTG curves of SiO_2_/SA hybrids compared to pure SA.

The TGA (Figure 8A) and DTG (Figure 8B) curves of the SiO_2_/SA hybrids showed altered thermal degradation behavior compared to pure SA. In all systems, an initial dehydration step (100–180 °C) was observed, amounting to 4.83% for SA, 1.91% for SiO_2_, and 2.83%, 1.62%, and 2.94% for SiO_2_/SA 2%, 5%, and 8%, respectively (Table 2). The decarboxylation process remained largely unaffected by the silica matrix, as evidenced by the similar positions of the DTG peak (~223–237 %/°C) for all hybrid samples. However, the 8% SA hybrid exhibited a slightly higher DTG peak (237.36 %/°C), suggesting a slightly enhanced thermal stability. This stabilization can be interpreted in the context of the reduced surface area of this sample (104.35 m^2^/g), compared to the higher values observed for SiO_2_/SA 2% (325.24 m^2^/g) and SiO_2_/SA 5% (138.69 m^2^/g). The lower surface area likely limited the exposure of the polymeric fraction to thermal flow, thus slowing degradation. Across all hybrids, the total weight loss was significantly reduced compared to pure SA, consistent with the lower polymer content. The Na_2_CO_3_ formation step (250–700 °C) was still evident, with weight losses increasing with SA content: 4.21%, 4.37%, and 6.90% for SiO_2_/SA 2%, 5%, and 8%, respectively. This trend reflects a more extensive carbonization process in hybrids with higher organic content. In contrast, pure SiO_2_ displayed a broad and continuous weight loss in the 180–700 °C range, attributed to progressive silanol condensation.

At higher temperatures (500–950 °C), pure SA showed a clear Na_2_CO_3_ decomposition step (30.81%, DTG peak at 786.78 °C). Among the hybrids, this event was completely absent for SiO_2_/SA 2% and 8%, whereas it was still detectable for SiO_2_/SA 5% (2.04%) [60]. This behavior suggests that the amount and distribution of residual sodium species strongly depend on the organic/inorganic balance. In the SiO_2_/SA 5% hybrid, the intermediate SA content provided sufficient sodium to form detectable Na_2_CO_3_, while the silica fraction was not high enough to fully encapsulate or immobilize these species. In contrast, the SiO_2_/SA 2% hybrid, due to its low organic fraction, generated only a limited amount of sodium, whereas in the SiO_2_/SA 8% hybrid, the stronger silica–sodium interactions and partial confinement within the denser silica matrix likely inhibited Na_2_CO_3_ decomposition under inert conditions. A summary of the main thermal events for pure SA and the hybrid materials is reported in Table 2.

### 2.7. Cytotoxicity Study

**Figure 9:**
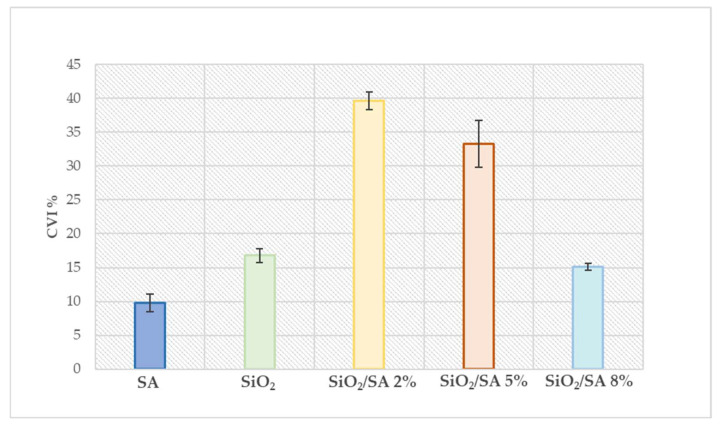
Cell viability inhibition of SiO_2_/SA hybrids against HaCaT cell line. CVI: cell viability inhibition.

A cytotoxicity assessment was conducted using HaCaT cells, an established model of healthy human keratinocytes, to evaluate the biocompatibility of hybrid SiO_2_/SA materials in comparison with their individual components, i.e., pure SiO_2_ and pure sodium alginate (SA) (Figure 9). Both references exhibited low cytotoxicity, with cell viability inhibition (CVI%) values remaining below 20%. Interestingly, incorporation of 2% SA into the silica matrix (SiO_2_/SA 2%) led to a marked increase in cytotoxicity, elevating CVI% to approximately 40%. This trend was reversed at higher SA loadings: SiO_2_/SA 5% showed a CVI% of about 33%, still consistent with a good biocompatibility [61], and SiO_2_/SA 8% returned to values comparable to pure SiO_2_, around 15%. These results suggest that the cytotoxicity of the hybrids is not simply governed by the presence of SA, which is known to be biocompatible, but rather by the physicochemical changes introduced upon hybrid formation. BET analysis revealed that SiO_2_/SA 2% possessed a specific surface area nearly three times greater than that of pure SiO_2_, which may account for the enhanced interaction with cell membranes and the observed cytotoxicity. As the SA content increased, the surface area decreased accordingly, paralleling the drop in cytotoxic effects. This was also because of the anti-inflammatory effect of SA [62]. Furthermore, these findings are consistent with previous literature showing that an increased surface area in nanostructured materials can amplify cell–material interactions, leading to higher biological reactivity. As demonstrated by Spyrogianni et al. (2017), both particle size and surface area are critical determinants of silica nanoparticle cytotoxicity [63]. Overall, the data indicate that tuning the SA content in SiO_2_-based hybrids allows modulation of cytotoxicity, with intermediate compositions (e.g., 2–5%) requiring careful evaluation due to their heightened surface reactivity.

## 3. Materials and Methods

### 3.1. Sol–Gel Synthesis

**Figure 10:**
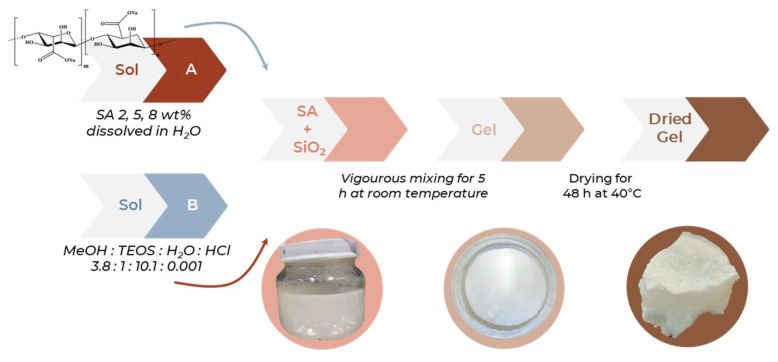
Sol–gel synthesis of SiO_2_/SA hybrids. SA: sodium alginate; TEOS: tetraethyl orthosilicate; MeOH: methanol.

SiO_2_ and SiO_2_/SA hybrid materials containing 2, 5, and 8% of SA were synthesized via a sol–gel route. SA was purchased from Thermo Fisher Scientific (Waltham, MA, USA; Product number A18565, Lot 10242904), characterized by a low viscosity of 9 mPa·s (1% solution at 20 °C). The required amounts of SA, corresponding to 2, 5, or 8% by weight relative to the final SiO_2_ mass, were dissolved in deionized water and slowly added to the solution in a beaker placed on a magnetic stirrer at 40 °C. For the pure SiO_2_ sample, no SA was added. Tetraethyl orthosilicate (TEOS, Si(OC_2_H_5_)_4_, Sigma-Aldrich, St. Louis, MO, USA) was used as the silica precursor and dissolved in methanol (≥99.8%, HiPerSolv CHROMANORM^®^, gradient grade for HPLC, VWR; purchased from Sigma-Aldrich), which served as a co-solvent. Hydrochloric acid (HCl, 37% *w*/*w*, CAS No. 7647-01-0, MFCD00011324) was obtained from Sigma-Aldrich Fine Chemicals Biosciences (Product code H1758100ML, 100 mL). The SA solution was added dropwise to the TEOS/methanol mixture to obtain a stable, white, opalescent mixture free of visible aggregates or phase separation. The molar ratios employed for all samples were as follows: TEOS:SiO_2_ = 1; H_2_O:TEOS = 10.1; MeOH:TEOS = 3.8. The final sol was stirred at room temperature for 5 h. After stirring, the system was sealed to allow gelation. The resulting gels were dried in a static oven at 50 °C until complete solvent removal. The dried materials were then stored in airtight containers for further characterization (Figure 10).

### 3.2. FTIR Analysis

FTIR spectra were recorded using the Prestige21 Shimadzu system, equipped with a DTGS KBr detector. A resolution of 2 cm^−1^ was used, and 60 scans were acquired within the range of 400–4000 cm^−1^. Firstly, 1:100 = sample:KBr disks were prepared for analysis. FTIR spectra were elaborated using IRsolution (version 1.60, Shimadzu, Milan, Italy) and Origin 8 (version 2022b, OriginLab Corporation, Northampton, MA, USA) softwares. The spectra of pure silica and pure sodium alginate were used as comparisons.

### 3.3. XRD Analysis

A Philips X’Pert PRO diffractometer (now part of Malvern PANAlytical, Malvern, UK) equipped with a fast detector (X’Celerator) was used for the XRD investigations. Measurements were carried out at room temperature with continuous sweep, using Cu-kα radiation (λ = 1.5405 Å), with a voltage of 40 kV and a current of 40 mA, in the 2θ range from 5 to 70° with a step of 0.04° and a time per step of 500 s.

### 3.4. BET Study

The surface area was determined by BET analysis (Micromeritics GEMINI V, Norcross, GA, USA). All samples were pre-treated with nitrogen at 80 °C for 24 h. This temperature was selected to facilitate the evaporation of any pollutants present on the surface of the particles.

### 3.5. Moisture Absorption Analysis

The hybrid SiO_2_/SA, SiO_2_, and SA samples were first ground and then dried under vacuum for 24 h to completely remove any residual moisture. Each sample was then individually placed inside a vacuum chamber maintained at a controlled relative humidity of 99% using a thermostat-regulated system. The sample weight was recorded at regular intervals: every hour for the first 8 h, and subsequently at 24 and 48 h. The experiment was carried out separately for each sample to avoid interactions between materials and to ensure consistent humidity conditions within the chamber.

### 3.6. Thermal Analysis

The thermal analyses of SiO_2_/SA hybrids were performed through simultaneous DSC/TGA with the Discovery SDT 650. The materials were ground before starting the analysis. Approximately 10 mg of each sample was placed in a 90 µL alumina crucible. Prior to TGA analysis, all samples were stabilized through an isothermal treatment at 30 °C for 20 min in order to remove physical absorbed water. The flow rate applied for the test was 100 mL/min with a heating rate of 5 °C/min, starting from 100 °C to 1000 °C in both hybrids and references (SA and SiO_2_), as reported by Fiorentino et al. 2024 [64]. Measurements were elaborated using TRIOS 5.1.1 (build 46572) software by TA Instruments (New Castle, DE, USA) and Origin 8 software.

### 3.7. Cytotoxicity Study

Human keratinocyte cell line (HaCaT) was cultured at 37 °C in a humidified incubator with 5% CO_2_, using Dulbecco’s Modified Eagle Medium (DMEM) supplemented with 10% fetal bovine serum, 50 U/mL penicillin, and 100 μg/mL streptomycin [65]. Cells were seeded into 96-well plates at a density of 2.0 × 10^4^ cells per well and exposed to 1 mg of the test materials for 24 h [66]. Cell viability inhibition (CVI%) was evaluated using the MTT assay, which measures mitochondrial dehydrogenase activity, serving as an indirect indicator of potential cytotoxic effects through changes in cellular metabolic activity. Each experimental condition was tested in triplicate in independent assays. Data are reported as mean ± standard deviation.

## 4. Conclusions

This study demonstrated how the incorporation of different percentages of sodium alginate (SA) into an amorphous silica s(SiO_2_) matrix via sol–gel synthesis significantly influenced the physico-chemical and biological properties of the resulting hybrid materials. BET and moisture absorption analyses revealed that SiO_2_/SA 2% showed the highest surface area and absorption capacity, attributed to the formation of a more open and porous structure induced by the polymer network. As the SA content increased to 5% and 8%, the surface area progressively decreased (138.7 and 104.3 m^2^/g, respectively), although it was still much higher than that of pure SiO_2_ (9.86 m^2^/g), likely due to partial pore blocking by the polymer network. Cytotoxicity tests on HaCaT cells revealed that the higher surface area of the 2% SA hybrid correlated with higher cytotoxicity (~40% CVI), likely due to greater interactions with cell membranes.

In contrast, cytotoxicity decreased with increasing SA content. The SiO_2_/SA 5% hybrid showed a CVI of 33%, which is considered non-cytotoxic, while SiO_2_/SA 8% hybrid exhibited the lowest cytotoxicity (~15% CVI), comparable to pure SA and SiO_2_. Although the 8% hybrid demonstrated excellent biocompatibility, its lower surface area and FTIR spectral features suggested a less homogeneous structure, possibly due to the physical entrapment of excess SA that did not chemically interact with the silica matrix. Considering the balance between physico-chemical properties and biological response, SiO_2_/SA5% represented the most promising formulation, combining good functional performance with acceptable biocompatibility. These results highlight that further optimization with intermediate SA concentrations (e.g., 6–7%) could further reduce cytotoxicity below the 30% threshold while preserving desirable properties, supporting the potential of SiO_2_/SA 5% hybrids for future biomedical applications such as controlled drug delivery and tissue regeneration.

## Figures and Tables

**Table 1:** BET surface areas of all prepared samples after treatment at 80 °C for 24 h.

Samples	BET Surface Area (m^2^/g)
SA	0.23 ± 0.0067
SiO_2_	9.69 ± 0.36
SiO_2_/SA 2%	325.24 ± 5.33
SiO_2_/SA 5%	138.69 ± 2.12
SiO_2_/SA 8%	104.35 ± 2.09

**Table 2:** Summary table of SiO_2_/SA hybrids and SA thermal events, weight loss, and DTG values.

			Weight Loss (%)/DTG (%/°C)			
T_range_/°C	Phenomena	SA	SiO_2_	SiO_2_/SA 2%	SiO_2_/SA 5%	SiO_2_/SA 8%
100–180	1. Dehydration	4.83%	1.91%	2.83%	1.62%	2.94%
180–250	2a. Decarboxylation (Exo)	40.34%, 234.78 %/°C	-	1.37% 223.01%/°C	2.85% 224.91 %/°C	3.28% 237.36 %/°C
180–700	2b. Silanol condensation (Endo)	-	5.13%	-	-	-
250–500 (SA and SiO_2_/SA 5%)/ 250–700 (SiO_2_/SA 2 and 8%)	3. Na_2_CO_3_ formation (Exo)	13.40% 417.37 %/°C	-	4.18	4.47%	6.90%
500–950	4. Na_2_CO_3_ decomposition (Endo)	30.81% 786.78%/°C	-	-	1.95%	-

## Data Availability

The original contributions presented in this study are included in the article. Further inquiries can be directed to the corresponding author(s).

## References

[1] Langer R., Vacanti J.P. (1993). Tissue Engineering. Science.

[2] He X., Han Z., Ruan Y., Wang Z., Liao B., Li X., Tan J., Han X., Shen J., Bai D. (2025). Smart Responsive Biomaterials for Spatiotemporal Modulation of Functional Tissue Repair. Mater. Today Bio..

[3] Baskaran P., Muthiah B., Uthirapathy V. (2025). A Systematic Review on Biomaterials and Their Recent Progress in Biomedical Applications: Bone Tissue Engineering. Rev. Inorg. Chem..

[4] Ajmal S., Athar Hashmi F., Imran I. (2022). Recent Progress in Development and Applications of Biomaterials. Mater. Today Proc..

[5] Liu X., Ma P.X. (2004). Polymeric Scaffolds for Bone Tissue Engineering. Ann. Biomed. Eng..

[6] Deshmukh K., Kovářík T., Křenek T., Docheva D., Stich T., Pola J. (2020). Recent Advances and Future Perspectives of Sol–Gel Derived Porous Bioactive Glasses: A Review. RSC Adv..

[7] Vijayakumar N., Venkatraman S.K., Soundhariyaa T.N., Mohan S., Magesvaran M.K., Genasan K., Alex R.A., Abraham J., Swamiappan S. (2025). Fuel-assisted sol-gel combustion synthesis of monticellite: Structural, mechanical, and biological characterization for tissue engineering. J. Sci. Adv. Mater. Devices.

[8] Thapa R.K., Kiick K.L., Sullivan M.O. (2020). Encapsulation of Collagen Mimetic Peptide-Tethered Vancomycin Liposomes in Collagen-Based Scaffolds for Infection Control in Wounds. Acta Biomater..

[9] D’Angelo A., Fiorentino M., Viola V., Vertuccio L., Catauro M. (2024). Effect of Nitric Acid on the Synthesis and Biological Activity of Silica–Quercetin Hybrid Materials via the Sol-Gel Route. Appl. Sci..

[10] Shoushtari M.S., Hoey D., Biak D.R.A., Abdullah N., Kamarudin S., Zainuddin H.S. (2024). Sol–Gel—templated Bioactive Glass Scaffold: A Review. Res. Biomed. Eng..

[11] Niari S.A., Rahbarghazi R., Geranmayeh M.H., Karimipour M. (2022). Biomaterials Patterning Regulates Neural Stem Cells Fate and Behavior: The Interface of Biology and Material Science. J. Biomed. Mater. Res..

[12] Raffaini G., Pirozzi P., Catauro M., D’Angelo A. (2024). Hybrid Organic–Inorganic Biomaterials as Drug Delivery Systems: A Molecular Dynamics Study of Quercetin Adsorption on Amorphous Silica Surfaces. Coatings.

[13] Pamula E., Kokoszka J., Cholewa-Kowalska K., Laczka M., Kantor L., Niedzwiedzki L., Reilly G.C., Filipowska J., Madej W., Kolodziejczyk M. (2011). Degradation, Bioactivity, and Osteogenic Potential of Composites Made of PLGA and Two Different Sol–Gel Bioactive Glasses. Ann. Biomed. Eng..

[14] Zdarta J., Jesionowski T. (2022). Silica and Silica-Based Materials for Biotechnology, Polymer Composites, and Environmental Protection. Materials.

[15] Dixit M., Mishra M., Joshi P.A., Shah D.O. (2013). Study on the Catalytic Properties of Silica Supported Copper Catalysts. Procedia Eng..

[16] Azizi L., Turkki P., Huynh N., Massera J.M., Hytönen V.P. (2021). Surface Modification of Bioactive Glass Promotes Cell Attachment and Spreading. ACS Omega.

[17] Chaturvedi K., Ganguly K., More U.A., Reddy K.R., Dugge T., Naik B., Aminabhavi T.M., Noolvi M.N. (2019). Sodium Alginate in Drug Delivery and Biomedical Areas. Natural Polysaccharides in Drug Delivery and Biomedical Applications.

[18] Ahmad Raus R., Wan Nawawi W.M.F., Nasaruddin R.R. (2021). Alginate and Alginate Composites for Biomedical Applications. Asian J. Pharm. Sci..

[19] Shah F.A., Thomsen P., Palmquist A. (2019). Osseointegration and Current Interpretations of the Bone-Implant Interface. Acta Biomater..

[20] Drago E., Campardelli R., Pettinato M., Perego P. (2020). Innovations in Smart Packaging Concepts for Food: An Extensive Review. Foods.

[21] De Paula J.H., Quevedo B.V., Komatsu D., Santos A.R., De Souza A.L., De Rezende Duek E.A. (2025). Development of Silica/Collagen Hybrids Synthesized Via a Simplified Sol-Gel Reaction for Biomaterial Applications. Silicon.

[22] Maleki H., Shahbazi M.-A., Montes S., Hosseini S.H., Eskandari M.R., Zaunschirm S., Verwanger T., Mathur S., Milow B., Krammer B. (2019). Mechanically Strong Silica-Silk Fibroin Bioaerogel: A Hybrid Scaffold with Ordered Honeycomb Micromorphology and Multiscale Porosity for Bone Regeneration. ACS Appl. Mater. Interfaces.

[23] Abka-khajouei R., Tounsi L., Shahabi N., Patel A.K., Abdelkafi S., Michaud P. (2022). Structures, Properties and Applications of Alginates. Mar. Drugs.

[24] Guo X., Wang Y., Qin Y., Shen P., Peng Q. (2020). Structures, Properties and Application of Alginic Acid: A Review. Int. J. Biol. Macromol..

[25] Zhang X., Huang C., Zhao Y., Jin X. (2017). Preparation and Characterization of Nanoparticle Reinforced Alginate Fibers with High Porosity for Potential Wound Dressing Application. RSC Adv..

[26] Abdelhamid M.A.A., Khalifa H.O., Ki M.-R., Pack S.P. (2024). Nanoengineered Silica-Based Biomaterials for Regenerative Medicine. Int. J. Mol. Sci..

[27] Lee Y.-G., Park J.-H., Oh C., Oh S.-G., Kim Y.C. (2007). Preparation of Highly Monodispersed Hybrid Silica Spheres Using a One-Step Sol−Gel Reaction in Aqueous Solution. Langmuir.

[28] Marangoni Júnior L., Da Silva R.G., Anjos C.A.R., Vieira R.P., Alves R.M.V. (2021). Effect of Low Concentrations of SiO2 Nanoparticles on the Physical and Chemical Properties of Sodium Alginate-Based Films. Carbohydr. Polym..

[29] Marangoni Júnior L., Fozzatti C.R., Jamróz E., Vieira R.P., Alves R.M.V. (2022). Biopolymer-Based Films from Sodium Alginate and Citrus Pectin Reinforced with SiO_2_. Materials.

[30] Ashour M.M., Mabrouk M., Soliman I.E., Beherei H.H., Tohamy K.M. (2021). Mesoporous Silica Nanoparticles Prepared by Different Methods for Biomedical Applications: Comparative Study. IET Nanobiotechnol..

[31] Thakur S., Arotiba O.A. (2018). Synthesis, Swelling and Adsorption Studies of a pH-Responsive Sodium Alginate–Poly(Acrylic Acid) Superabsorbent Hydrogel. Polym. Bull..

[32] Dalal S.R., Hussein M.H., El-Naggar N.E.-A., Mostafa S.I., Shaaban-Dessuuki S.A. (2021). Characterization of Alginate Extracted from Sargassum Latifolium and Its Use in Chlorella Vulgaris Growth Promotion and Riboflavin Drug Delivery. Sci. Rep..

[33] Salisu A., Sanagi M.M., Abu Naim A., Abd Karim K.J., Wan Ibrahim W.A., Abdulganiyu U. (2016). Alginate Graft Polyacrylonitrile Beads for the Removal of Lead from Aqueous Solutions. Polym. Bull..

[34] Onbas R., Yesil-Celiktas O. (2019). Synthesis of Alginate-silica Hybrid Hydrogel for Biocatalytic Conversion by Β-glucosidase in Microreactor. Eng. Life Sci..

[35] Tabish M.S., Hanapi N.S.M., Ibrahim W.N.W., Saim N., Yahaya N. (2019). Alginate-Graphene Oxide Biocomposite Sorbent for Rapid and Selective Extraction of Non-Steroidal Anti-Inflammatory Drugs Using Micro-Solid Phase Extraction. Indones. J. Chem..

[36] Derkach S.R., Voron’ko N.G., Sokolan N.I., Kolotova D.S., Kuchina Y.A. (2020). Interactions between Gelatin and Sodium Alginate: UV and FTIR Studies. J. Dispers. Sci. Technol..

[37] Pannier A., Soltmann U., Soltmann B., Altenburger R., Schmitt-Jansen M. (2014). Alginate/Silica Hybrid Materials for Immobilization of Green Microalgae Chlorella Vulgaris for Cell-Based Sensor Arrays. J. Mater. Chem. B.

[38] Fiorentino M., Piccolella S., Gravina C., Stinca A., Esposito A., Catauro M., Pacifico S. (2022). Encapsulating Calendula Arvensis (Vaill.) L. Florets: UHPLC-HRMS Insights into Bioactive Compounds Preservation and Oral Bioaccessibility. Molecules.

[39] Kebede G.G., Mitev P.D., Briels W.J., Hermansson K. (2018). Red-Shifting and Blue-Shifting OH Groups on Metal Oxide Surfaces–towards a Unified Picture. Phys. Chem. Chem. Phys..

[40] Jiang X., Tang X., Tang L., Zhang B., Mao H. (2019). Synthesis and Formation Mechanism of Amorphous Silica Particles via Sol–Gel Process with Tetraethylorthosilicate. Ceram. Int..

[41] De Los Arcos T., Müller H., Wang F., Damerla V.R., Hoppe C., Weinberger C., Tiemann M., Grundmeier G. (2021). Review of Infrared Spectroscopy Techniques for the Determination of Internal Structure in Thin SiO2 Films. Vib. Spectrosc..

[42] Wiercigroch E., Szafraniec E., Czamara K., Pacia M.Z., Majzner K., Kochan K., Kaczor A., Baranska M., Malek K. (2017). Raman and Infrared Spectroscopy of Carbohydrates: A Review. Spectrochim. Acta Part A Mol. Biomol. Spectrosc..

[43] Villacrés N.A., Cavalheiro E., Schmitt C., Venâncio T., Alarcón H., Valderrama A. (2023). Preparation of Composite Films of Sodium Alginate-Based Extracted from Seaweeds Macrocystis Pyrifera and Lessonia Trabeculata Loaded with Aminoethoxyvinylglycine. J. Braz. Chem. Soc..

[44] Jyoti Borah S., Kumar R., Prasad Singh P., Kumar V. (2024). SnO_2_ Encapsulated in Alginate Matrix: Evaluation and Optimization of Bioinspired Nanoadsorbents for Azo Dye Removal. ChemBioChem.

[45] Sundarrajan P., Eswaran P., Marimuthu A., Subhadra L.B., Kannaiyan P. (2012). One Pot Synthesis and Characterization of Alginate Stabilized Semiconductor Nanoparticles. Bull. Korean Chem. Soc..

[46] Mortalò C., Russo P., Miorin E., Zin V., Paradisi E., Leonelli C. (2023). Extruded Composite Films Based on Polylactic Acid and Sodium Alginate. Polymer.

[47] Paradisi E., Mortalò C., Russo P., Zin V., Miorin E., Montagner F., Leonelli C., Deambrois S.M. (2024). Facile and Effective Method for the Preparation of Sodium Alginate/TiO_2_ Bio-Composite Films for Different Applications. Macromol. Symp..

[48] Skwira A., Szewczyk A., Konopacka A., Górska M., Majda D., Sądej R., Prokopowicz M. (2019). Silica-Polymer Composites as the Novel Antibiotic Delivery Systems for Bone Tissue Infection. Pharmaceutics.

[49] Ebnalwaled A.A., Sadek A.H., Ismail S.H., Mohamed G.G. (2022). Structural, Optical, Dielectric, and Surface Properties of Polyimide Hybrid Nanocomposites Films Embedded Mesoporous Silica Nanoparticles Synthesized from Rice Husk Ash for Optoelectronic Applications. Opt. Quant. Electron..

[50] Han X., Liang J., Fukuda S., Zhu L., Wang S. (2022). Sodium Alginate–Silica Composite Aerogels from Rice Husk Ash for Efficient Absorption of Organic Pollutants. Biomass Bioenergy.

[51] Qi Y., Li J., Chen Y., Zhu B., Zhou X., Xiao X., Gu Z., Qian J., He C., Lai M. (2025). Differential Fiber Optic Humidity Sensor Based on Superhydrophilic SiO2/Polyethylene Glycol Composite Film with Linear Response. Opt. Fiber Technol..

[52] Zhang D., Ding J., Zhou Y., Ju J. (2024). Research Progress on Moisture-Sorption Actuators Materials. Nanomaterials.

[53] Gómez Vargas C., Ponce N.M.A., Stortz C.A., Fissore E.N., Bonelli P., Otálora González C.M., Gerschenson L.N. (2025). Pectin Obtention from Agroindustrial Wastes of Malus Domestica Using Green Solvents (Citric Acid and Natural Deep Eutectic Solvents). Chemical, Thermal, and Rheological Characterization. Front. Chem..

[54] Akshaya S., Nathanael A.J. (2024). A Review on Hydrophobically Associated Alginates: Approaches and Applications. ACS Omega.

[55] Hernández-González A.C., Téllez-Jurado L., Rodríguez-Lorenzo L.M. (2021). Preparation of Covalently Bonded Silica-Alginate Hybrid Hydrogels by SCHIFF Base and Sol-Gel Reactions. Carbohydr. Polym..

[56] Flores-Hernández C.G., Cornejo-Villegas M.D.L.A., Moreno-Martell A., Del Real A. (2021). Synthesis of a Biodegradable Polymer of Poly (Sodium Alginate/Ethyl Acrylate). Polymers.

[57] Dos Santos Araújo P., Belini G.B., Mambrini G.P., Yamaji F.M., Waldman W.R. (2019). Thermal Degradation of Calcium and Sodium Alginate: A Greener Synthesis towards Calcium Oxide Micro/Nanoparticles. Int. J. Biol. Macromol..

[58] Liu L., Lu Y., Qiu D., Wang D., Ding Y., Wang G., Liang Z., Shen Z., Li A., Chen X. (2022). Sodium Alginate-Derived Porous Carbon: Self-Template Carbonization Mechanism and Application in Capacitive Energy Storage. J. Colloid Interface Sci..

[59] Soares J.D.P., Dos Santos J.E., Chierice G.O., Cavalheiro É.T.G. (2004). Thermal Behavior of Alginic Acid and Its Sodium Salt. Eclet. Quim..

[60] Wei J., Yang S., Zhu Z., Lu J., Zhang B., Zhang M., Wei W. (2025). Low-Temperature Dried Alginate/Silica Hybrid Aerogel Beads with Tunable Surface Functionalities for Removal of Lead Ions from Water. Gels.

[61] López-García J., Lehocký M., Humpolíček P., Sáha P. (2014). HaCaT Keratinocytes Response on Antimicrobial Atelocollagen Substrates: Extent of Cytotoxicity, Cell Viability and Proliferation. J. Funct. Biomater..

[62] Fernando I.P.S., Jayawardena T.U., Sanjeewa K.K.A., Wang L., Jeon Y.-J., Lee W.W. (2018). Anti-Inflammatory Potential of Alginic Acid from Sargassum Horneri against Urban Aerosol-Induced Inflammatory Responses in Keratinocytes and Macrophages. Ecotoxicol. Environ. Saf..

[63] Spyrogianni A., Sotiriou G.A., Brambilla D., Leroux J.-C., Pratsinis S.E. (2017). The Effect of Settling on Cytotoxicity Evaluation of SiO_2_ Nanoparticles. J. Aerosol Sci..

[64] Fiorentino M., D’Angelo A., Vertuccio L., Khan H., Catauro M. (2024). Characterization of Grape Extract-Colored SiO2 Synthesized via the Sol–Gel Method. Appl. Sci..

[65] Piccolella S., Fiorentino M., Cimmino G., Esposito A., Pacifico S., Cilentan Cichorium Intybus L. (2024). Organs: UHPLC-QqTOF-MS/MS Analysis for New Antioxidant Scenario, Exploitable Locally and Beyond. Future Foods.

[66] Brahmi-Chendouh N., Piccolella S., Gravina C., Fiorentino M., Formato M., Kheyar N., Pacifico S. (2022). Ready-to-Use Nutraceutical Formulations from Edible and Waste Organs of Algerian Artichokes. Foods.

